# Elevational Gradients in β-Diversity Reflect Variation in the Strength of Local Community Assembly Mechanisms across Spatial Scales

**DOI:** 10.1371/journal.pone.0121458

**Published:** 2015-03-24

**Authors:** J. Sebastián Tello, Jonathan A. Myers, Manuel J. Macía, Alfredo F. Fuentes, Leslie Cayola, Gabriel Arellano, M. Isabel Loza, Vania Torrez, Maritza Cornejo, Tatiana B. Miranda, Peter M. Jørgensen

**Affiliations:** 1 Center for Conservation and Sustainable Development, Missouri Botanical Garden, P.O. Box 299, St. Louis, Missouri 63166, United States of America; 2 Escuela de Biología, Pontificia Universidad Católica del Ecuador, Av. 12 de Octubre 1076 y Roca, Apdo. 17-01-2184, Quito, Ecuador; 3 Department of Biology, Washington University, St. Louis, Missouri 63130, United States of America; 4 Departamento de Biología, Área de Botánica, Universidad Autónoma de Madrid, Calle Darwin 2, 28049 Madrid, Spain; 5 Herbario Nacional de Bolivia, Campus Universitario Cota-Cota, calle 27, Correo Central Cajón Postal 10077, La Paz, Bolivia; 6 Real Jardín Botánico, Consejo Superior de Investigaciones Científicas, Plaza de Murillo 2, 28014 Madrid, Spain; 7 Missouri Botanical Garden, P.O. Box 299, St. Louis, Missouri 63166, United States of America; 8 Department of Biology, University of Missouri, St. Louis, Missouri 63121, United States of America; 9 Division of Plant Conservation and Population Biology, Department of Biology, University of Leuven, B-3001 Leuven, Belgium; Berkeley, UNITED STATES

## Abstract

Despite long-standing interest in elevational-diversity gradients, little is known about the processes that cause changes in the compositional variation of communities (β-diversity) across elevations. Recent studies have suggested that β-diversity gradients are driven by variation in species pools, rather than by variation in the strength of local community assembly mechanisms such as dispersal limitation, environmental filtering, or local biotic interactions. However, tests of this hypothesis have been limited to very small spatial scales that limit inferences about how the relative importance of assembly mechanisms may change across spatial scales. Here, we test the hypothesis that scale-dependent community assembly mechanisms shape biogeographic β-diversity gradients using one of the most well-characterized elevational gradients of tropical plant diversity. Using an extensive dataset on woody plant distributions along a 4,000-m elevational gradient in the Bolivian Andes, we compared observed patterns of β-diversity to null-model expectations. β-deviations (standardized differences from null values) were used to measure the relative effects of local community assembly mechanisms after removing sampling effects caused by variation in species pools. To test for scale-dependency, we compared elevational gradients at two contrasting spatial scales that differed in the size of local assemblages and regions by at least an order of magnitude. Elevational gradients in β-diversity persisted after accounting for regional variation in species pools. Moreover, the elevational gradient in β-deviations changed with spatial scale. At small scales, local assembly mechanisms were detectable, but variation in species pools accounted for most of the elevational gradient in β-diversity. At large spatial scales, in contrast, local assembly mechanisms were a dominant force driving changes in β-diversity. In contrast to the hypothesis that variation in species pools alone drives β-diversity gradients, we show that local community assembly mechanisms contribute strongly to systematic changes in β-diversity across elevations. We conclude that scale-dependent variation in community assembly mechanisms underlies these iconic gradients in global biodiversity.

## Introduction

Changes in biological diversity along elevational gradients represent one of the most striking and consistent patterns of life on Earth [1–3]. Elevational-diversity gradients have puzzled biologists for centuries, but mechanisms responsible for them remain a source of contention, and a major focus of macroecological research [4,5]. Understanding the causes of elevational-diversity gradients not only represents one of the most classic and fundamental problems in ecology and evolution [1], but also has critical implications for the conservation and management of biodiversity in the face of anthropogenically driven global change [3,6].

Despite widespread interest in the causes of elevational-diversity gradients, empirical studies to date have focused almost exclusively on patterns of species richness [2,7]. In contrast, surprisingly little is known about the patterns and causes of spatial variation in community composition (β-diversity) across elevations. β-diversity is a critical component of biodiversity that reflects variation in species composition among local assemblages, as well as the relationship between local (α-) and regional (γ-) diversity [8–11]. Consequently, patterns of β-diversity can be used to study mechanisms of community assembly along environmental or geographic gradients [10]. At global scales, β-diversity has been shown to vary across latitudes, decreasing from tropical to temperate regions [12,13,11]. In contrast, we lack rigorous evaluations of elevational gradients in β-diversity. β-diversity has been reported to decrease towards high elevations [11]. However, the few reports of how β-diversity changes with elevation are typically limited by low replication [11,14] and/or short elevational extents [15], frequently lack the within-elevation replication necessary for measuring β-diversity at a particular point along the gradient [16,17], or are conducted only at very small spatial scales [11,14]. As a result, despite decades of research on elevational-diversity gradients and the important insights that can be gained from studying β-diversity, both the patterns and causes of elevational gradients in β-diversity remain largely unknown.

Multiple processes at various scales can cause variation in β-diversity. On the one hand, β-diversity is hypothesized to reflect community assembly mechanisms that selectively limit the membership and abundance of species in communities [14,18]. For example, changes in β-diversity can result from variation in the strength of dispersal limitation [19], species-sorting due to environmental heterogeneity [20], or priority effects [21]. On the other hand, changes in β-diversity are hypothesized to reflect variation in the characteristics of regional species pools [10,22–25]. For example, simulations have demonstrated that when the size of the species pool varies strongly among regions, random sampling alone can lead to differences in β-diversity: large species pools produce dissimilar local assemblages and high β-diversity, whereas small species pools produce similar local assemblages and low β-diversity [11]. Indeed, two recent studies of woody plant β-diversity along a latitudinal [11,26] and an elevational [11] gradient found that gradients in β-diversity disappeared after controlling for variation in species pools, a pattern which could suggest an overriding influence of broad-scale evolutionary and ecological processes responsible for the formation of regional species pools [11,24,26]. In contrast, other studies have found that gradients in β-diversity persist after controlling for variation in species pools [14,18], suggesting an important role for geographic variation in local community assembly mechanisms [27,28]. These conflicting patterns highlight the need for an expanded framework that explicitly considers the factors that would cause the relative importance of species pools and assembly processes to vary across biogeographic gradients [29].

One key factor that may influence variation in β-diversity and its underlying mechanisms is spatial scale [2,30–33]. Spatial scale can strongly influence both patterns [34,35] and mechanisms [35,36] of β-diversity. For example, increasing the size of regions and/or the geographic distances among local assemblages can increase the relative importance of local processes by increasing environmental heterogeneity that would lead to stronger species sorting, or by increasing isolation and dispersal limitation [37]. In contrast, local deterministic processes might be weak when local assemblages are small [38], so sampling effects and variation in species pools might become the overriding force behind β-diversity gradients at small scales. To date, however, elevational studies of β-diversity have not explicitly examined the influence of spatial scale as a driver of biogeographic gradients in β-diversity and their underlying processes [11,14,18,26]. To the extent that mechanisms of community assembly vary with spatial scale [36,39,40], this could help reconcile contrasting patterns of β-diversity observed across elevational-diversity gradients.

In this study, we use a null-model approach to disentangle the scale-dependent contributions of local community assembly mechanisms and variation in regional species pools to elevational gradients in β-diversity. We present an analysis of a comprehensive study of tropical plant diversity along an elevational gradient in the Bolivian Andes. In contrast to previous null-model analyses based on a relatively small number of samples (7–8 plots), species (∼60–600), and short elevational extents (∼1200–2200 m) [11,14], we compared patterns of β-diversity along a 4,000-m elevational gradient that included 440 plots and 2,668 woody plant species. Importantly, our data set allowed us to test for scale-dependency by comparing patterns at two contrasting spatial scales that differed by at least an order of magnitude in the size of local assemblages and regions, as well as in the average distance among local assemblages. We compared observed elevational gradients in β-diversity to gradients expected by two null models of random assembly from regional species pools. If biogeographic variation in local community assembly mechanisms is not an important determinant of β-diversity gradients, then the elevational gradient in β-diversity should disappear after accounting for sampling effects and variation in species pools [11]. In contrast, if elevational changes in local assembly mechanisms are important, then elevational gradients in β-diversity should persist after removing the effects of variation in species pools. In contrast to the hypothesis that variation in species pools is the sole driver of gradients in β-diversity [11,26], we show that biogeographic differences in local assembly mechanisms contribute to a mid-elevational peak in β-diversity. Moreover, we find that this pattern is strongly scale-dependent and becomes stronger at larger spatial scales. Our results suggest that scale-dependent variation in community assembly mechanisms underlie these iconic gradients in global biodiversity.

## Materials and Methods

### The Madidi Project: A floristic inventory of northwestern Bolivia

**Fig 1:**
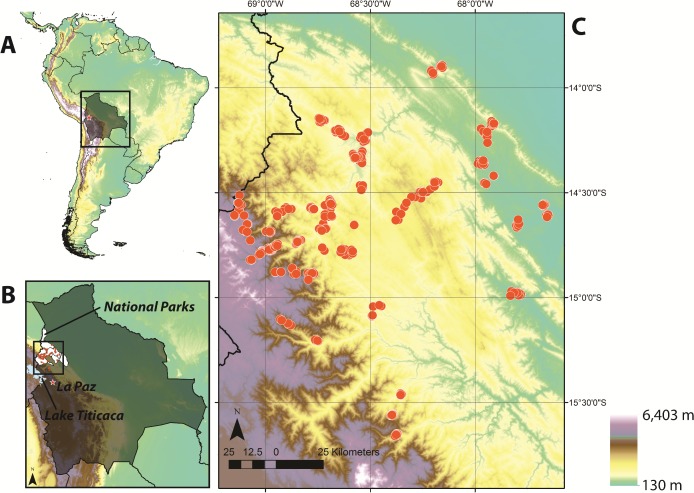
Regional network of forest plots sampled as part of The Madidi Project, a floristic inventory of northwestern Bolivia. The map shows the locations of 440 0.1-ha plots along a 4000-m elevational gradient in the Andes. A) Study region in Bolivia. B) and C) Distribution of plots along the eastern slopes of the Andes (∼250–4,350 m) in and around the multiple protected areas that form part of the Madidi region. Elevation data from WorldClim (www.worldclim.org); country borders from the GADM database (gadm.org).

Data used in these analyses were collected as part of the Madidi Project (www.mobot.org/madidi), a 12-year collaboration to study the flora in and around Madidi National Park, Bolivia (Fig. 1) [41]. The Madidi region encompasses a wide range of environmental conditions and vegetation types [42], extending from lowland plains at ∼200 m to mountain peaks above 6,000 m. Species composition and abundance of woody plants were obtained from 440 0.1-ha (20×50-m) plots (Fig. 1). Plots were generally located in closed-canopy mature forest at least 100 m from one another (average nearest neighbor distance = ∼540 m). Plots range in elevation from 254 to 4,351 m, covering the entire elevational distribution of forests in the eastern slopes of the Bolivian Andes. Each 0.1-ha plot was divided into ten 10×10-m subplots. Within each subplot, all woody plants with a diameter at breast height (130 cm) of at least 2.5 cm were measured and identified to a species or morphospecies name. Specimens were collected to voucher each species/morphospecies at each site; these specimens are deposited at the Missouri Botanical Garden and the Herbario Nacional de Bolivia. Fieldwork was conducted with permits granted by the Ministerio de Medio Ambiente y Agua of Bolivia. Extensive taxonomic work was conducted to standardize taxonomic names across all plots. Unidentified individuals (< 3.2%) were excluded from analyses. In total, our dataset contains information on the distribution of 159,040 individuals and 2,668 species/morphospecies. Plot data are deposited and can be accessed via Tropicos. Summary information for each small- and large-scale region can be found in the Supporting Information (S1 and S2 Datasets).

### Partitioning diversity into regional (γ-), local (α-) and β-components

For regions along the elevational gradient, we measured β-diversity by partitioning diversity (*D*) among its regional (γ-), local (α-) and β-components. Following Jost [43], the β-diversity component was defined as:

$${}^{q}D_{\beta }=\frac{{}^{q}D_{\gamma }}{{}^{q}D_{\alpha }}$$

where ^*q*^ *D*_*γ*_ is the regional diversity and ^*q*^ *D*_*α*_ the diversity of local assemblages. The mathematical definitions of ^*q*^ *D*_*γ*_ and ^*q*^ *D*_*α*_ can be found in Jost [43]. In this framework, *q* is a non-negative number that defines the “order” of the diversity components, and controls the sensitivity of the index to rare species. We partitioned diversity using components of order one (*q* = 1), which weigh species proportionally to their abundances, making ^*q*^ *D*_*γ*_ and ^*q*^ *D*_*α*_ equal to the exponential of Shannon diversity. Diversity partitioning was conducted in R using the package “vegetarian” [44].

To investigate whether our results are sensitive to changes in metric, we repeated our analyses using three additional measures of β-diversity: (1) mean of Bray-Curtis distances among all pairs of local assemblages, (2) ^*q*^ *D*_*β*_ when *q* = 0, which weighs all species equally irrespective of abundance, and (3) proportional species turnover ($\beta =1-{\bar{\alpha }}_{richness}/\gamma_{richness}$) [8,11]. Results based on these alternative metrics lead to similar conclusions (S1 Results). All β-diversity metrics used in our analyses represent “variation” *sensu* Anderson et al. [9], which is defined as the non-directional change in community composition across sampling units.

### Spatial scales of analysis

To test for scale-dependence in patterns of β-diversity, we defined local assemblages and regions using two contrasting spatial scales (hereafter referred to as “small” and “large”). At both scales, the elevational span of analysis was very similar: the ∼4,000-m elevational gradient across the Madidi region. However, the contrasting scales differed by an order of magnitude or more in the size and distances between local assemblages (i.e. grain size and lag, respectively), as well as in the size of regions (i.e. spatial extent).

At the small scale, we defined a local assemblage as a 10×10-m subplot, and a region as a 0.1-ha plot (10 assemblages per region; *N* = 440 regions). At this scale, β-diversity represents variation in species composition within a small plot [11,14,26]. At the large scale, we defined a local assemblage as a 0.1-ha plot, and a region as a group of 10 plots located at a similar elevation (10 assemblages per region; N = 18 regions) [45]. We produced 18 large-scale regions by dividing the elevational gradient into equal-sized elevational bands, and selecting 10 plots falling within each band. Plots were selected to ensure that large-scale regions were comparable along the elevational gradient (S1 Methods). The typical distance among local assemblages in large-scale regions was ∼19 km, and the typical range in elevation was ∼165 m (S1 Methods). At this scale, β-diversity represents variation in species composition among plots within a narrow elevational band.

We used these contrasting spatial scales to compare elevational patterns in β-diversity and their underlying mechanisms between (1) the very small scales used in recent studies [11,14,26] and (2) the larger scales that ecologists would typically use to define regions along broad-scale environmental gradients. We did not examine β-diversity at larger elevational extents (>165 m) because an increase in the spatial extent of the elevational bands would confound variation in community composition within elevations with species turnover among elevations [9].

Because sampling effort across elevational bands was standardized in terms of area (i.e., 10 0.1-ha plots), and because we used forest plots to produce estimates of species pools across regions (elevational bands), our measures of diversity in regional species pools represent relative diversity densities, rather than total diversity. This can bias our estimates of species-pool diversity in two ways. First, if there are gradients in the density of individuals per plot, elevational bands with more individuals might appear to have higher diversity than elevational bands with fewer individuals [46]. Second, because the total number of unobserved species within an elevational band is likely to vary along the elevational gradient, our standardized sampling might accurately estimate the species pool in low-diversity elevational bands, but underestimate the size of the species pool in high-diversity elevational bands [47]. Both effects could modify the patterns in γ-diversity that we report here. To evaluate the extent to which these biases may influence our results, we used (1) rarefaction to standardize sampling by numbers of individuals, and (2) various metrics of extrapolation to estimate the total number of species that would be expected if sampling of species pools would have been complete (S2 Methods). We found that although there is a gradient in the density of individuals, and our sampling underestimates the total number of species present at a particular elevation, the overall patterns of γ-diversity would remain the same if other approaches to estimate regional species pools would have been used (S2 Methods). Furthermore, the proportion of the total species pool that was sampled at each elevation varies little across most of the elevational gradient. This suggests that although we are underestimating γ-diversity, additional field surveys designed to sample entire species pools—an impractical endeavor in most hyperdiverse tropical regions—would likely lead to the same general conclusions we reach from our standardized estimates.

### Random-assembly null models and β-deviations

To disentangle the contribution of local community assembly mechanisms from sampling effects owing to variation in species pools, we compared observed β-diversity to values expected under two null models. Both null models account for regional sampling effects due to the size and structure of species pools, but eliminate local processes that determine the abundances and distributions of species across local assemblages. Thus, deviations from the null models can be used to quantify the relative effects of local community assembly mechanisms [10,11]. Null models, however, are only approximate tools, and results must be interpreted as “a ‘toe-in-the-door’ regarding mechanisms” [9]. Further studies, particularly replicated experiments, monitoring studies along biogeographic gradients [24], and studies that integrate information from other dimensions of community structure (e.g. phylogenetic and functional [48]), will be needed to confirm the conclusions supported by our analyses.

The effects of local community assembly mechanisms on β-diversity can be mediated by (1) non-random patterns in the distribution of species across communities (e.g. spatial aggregation or “clumping”), or (2) variation in the distribution of individuals across species (i.e. structure in the regional species abundance distribution [SAD]) [18,22,23]. To examine these mechanisms, we compared observed β-diversity to two different null models that eliminate either one or both of these types of local effects. Our two null models differ in the way randomization algorithms model the regional SAD when creating null local assemblages:

1. **Fixed regional SAD null model.** This null model eliminates effects of local assembly processes that constrain the membership of individuals in local communities, and that create patterns of intraspecific aggregation and interspecific co-occurrence [11,23,26]. In this null model, the species pool is defined as the observed number and abundances of species in a region [11]. In this way, the regional SAD is constrained to be the same in null and empirical datasets. Local assemblages are then created by randomly sampling individuals without replacement from the regional species pool. Deviations from this null model represent the influence of local processes that cause non-random distributions of species across communities.
2. **Random regional SAD null model.** This second null model eliminates effects of local assembly processes that not only constrain the membership of species in local communities, but also processes that structure regional species abundances [18,23]. In this null model, the species pool is defined only as the observed number of species in a region. Here, the regional SAD is randomized by re-assigning individuals to each species in the region with equal probability. Local assemblages are then produced by randomly sampling individuals without replacement from the regional species pool using the randomized SAD. Deviations from this null model represent the influence of local processes causing non-random patterns in the distribution of (1) species across communities and (2) individuals across species.

Previous applications of these types of null models have constrained randomizations so that empirical and null local assemblages have the same total number of individuals [14,26,49]. Arguably, however, the number of individuals in a local assemblage (i.e. community size) is also controlled by local processes, which these null models supposedly eliminate [11]. Here, we focus on an alternative approach that eliminates this constraint from the randomization algorithms. Analyses based on null models that constrain numbers of individuals in local assemblages lead to similar conclusions (S2 Results).

After null assemblages were produced by a particular null model, we partitioned diversity in the same way as we did for the empirical data. This produced a null value of β-diversity expected from (1) random sampling from the observed species pool and (2) the absence of local community assembly mechanisms. We implemented 1,999 iterations of each null model, producing a frequency distribution of null β-diversity values for each region. Based on this frequency distribution, we calculated a β-deviation (*sensu* [11]):

$$\beta_{dev_{i}}=\frac{\beta_{obs_{i}}-{\bar{\text{β}}}_{null_{i}}}{\sigma_{null_{i}}}$$

where ${\bar{\text{β}}}_{null_{i}}$ and $\sigma_{null_{i}}$ are the average and standard deviation of the frequency distribution of null values for region *i*. A β-deviation is a standardized measure of the difference between observed and null β-diversity, and can be interpreted as the relative effect of local assembly mechanisms on β-diversity (e.g. dispersal limitation, habitat filtering) after removing effects of sampling from observed species pools [10,29]. We produced β-deviations along the elevational gradient by repeating these calculations for all regions. R functions to produce null local assemblages and calculate β-deviations are provided in the Supporting Information (S1 Code).

### Statistical analyses

To test for elevational gradients in diversity, we regressed observed γ-, α- and β-diversity against elevation using ordinary least-squares models (OLS) [9]. Due to non-linearity in these relationships, we compared fits of linear, quadratic and cubic regressions and selected the regression model with the lowest corrected Akaike information criterion (AICc) [50]. Identical analyses were also conducted to characterize elevational gradients in mean null β-diversity and β-deviations. If variation in local assembly mechanisms influence elevational gradients in β-diversity, we would expect a significant relationship between β-deviations and elevation. On the other hand, if elevational gradients in β-diversity were solely the result of sampling effects owing to variation in species pools, then β-deviations should not be significantly related to elevation [11].

To test for scale dependency in the contribution of local community assembly mechanisms to elevational patterns of β-diversity, we compared the strength and shape of elevational gradients in β-deviations between the small and large spatial scales. The strength of the gradients was measured using adjusted R^2^ values (_adj._R^2^), whereas the shape was measured using standardized regression coefficients. To compare _adj._R^2^ values and regression coefficients between gradients, we created 99% confidence regions around their original estimates using cubic OLS regressions and non-parametric bootstrapping [51,52]. If confidence regions for different gradients do not overlap each other’s estimates, we concluded that gradients were significantly different in strength or shape. We used cubic OLS models so that regression coefficients would be comparable among elevational gradients. For these scale analyses, we used orthogonal polynomials to make coefficients independent from one another; we also centered and standardized all dependent and predictor variables to eliminate effects of magnitude [53]. Significant differences between scales would suggest that elevational patterns of local assembly mechanisms are scale dependent.

Finally, we tested for scale dependency in the strength of local mechanisms structuring assemblages irrespective of elevational patterns. First, we compared average log-transformed β-deviations against zero using four separate one-sample t-tests, one for each combination of spatial scale (small versus large) and null model (fixed SAD versus randomized SAD). A significant difference from zero would suggest that assemblages are not the result of random uncorrelated sampling from species pools [23], and that local processes are important in creating structure among assemblages within regions. Second, we compared the magnitude of log-transformed β-deviations between scales using a linear mixed-effects model where scale and null model were fixed effects, and region was a random effect. To maintain independence between levels of the factor “scale”, we conducted this analysis using only the 262 small-scale regions that were not part of any large-scale region. Differences between scales would suggest that, independently of changes with elevation, the importance of local mechanisms structuring assemblages vary with spatial scale.

## Results

**Fig 2:**
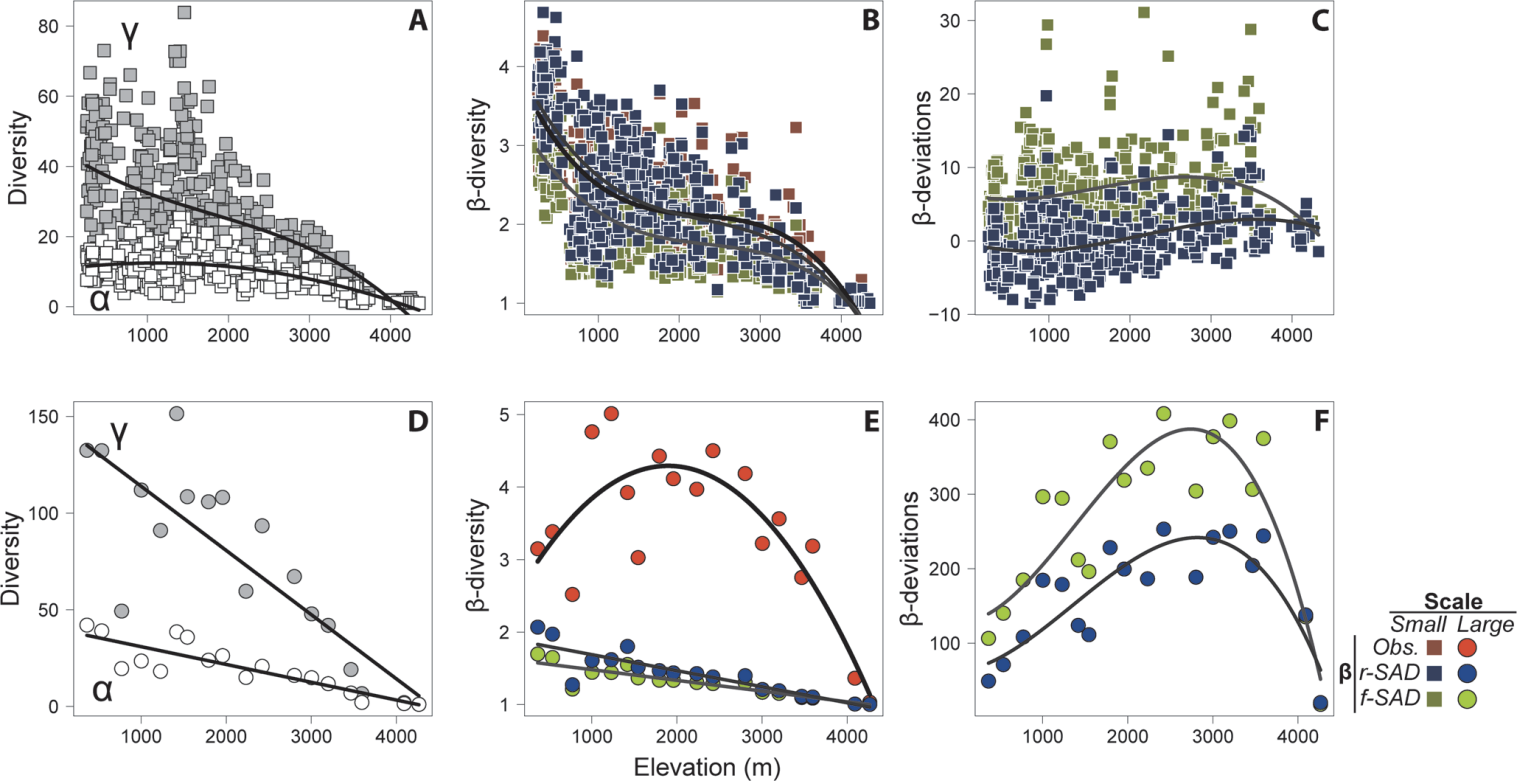
Elevational gradients in diversity at two contrasting spatial scales. Small (among 0.01-ha subplots within a 0.1-ha plot; top row) and large (among 0.1-ha plots within an elevational band; bottom row). A) and D) Regional (γ-) and local (α-) diversity. B) and E) Observed β-diversity and mean null β-diversity. C) and F) β-deviations (standardized effect sizes of β-diversity). Null β-diversity and β-deviations were calculated based on two null models, one that randomizes the regional species abundance distribution (r-SAD) and one that fixes it to be identical to the one observed in the empirical data (f-SAD; see Methods). Diversity was partitioned following Jost [43] and by weighting each species proportionally by its abundance (i.e. diversity of order 1). All relationships were statistically significant (Table 1).

Diversity varied strongly with elevation and spatial scale. At both small and large scales, γ- and α-diversity showed strong monotonic decreases with elevation (Table 1; Fig. 2). Observed β-diversity also varied strongly along the elevational gradient, and the shape and strength of the pattern differed between spatial scales. At the small scale, observed β-diversity had a moderate monotonically decreasing relationship with elevation. In contrast, at the large scale, β-diversity had a strong hump-shaped relationship with elevation, with a peak towards intermediate elevations (1,750–2,000 m), and a more pronounced decrease towards the highlands than toward the lowlands (Table 1; Fig. 2).

**Table 1:** Relationships between diversity and elevation.

**Spatial Scale**	**Diversity**	**Null Model**	_adj._ **R** ^2^	***p*-value**
Small	γ		0.39	< 0.001
	α		0.41	< 0.001
	β		0.54	< 0.001
	Mean Null β	r-SAD	0.58	< 0.001
	Mean Null β	f-SAD	0.55	< 0.001
	β-deviations	r-SAD	0.14	< 0.001
	β-deviations	f-SAD	0.08	< 0.001
Large	γ		0.72	< 0.001
	α		0.75	< 0.001
	β		0.73	< 0.001
	Mean Null β	r-SAD	0.77	< 0.001
	Mean Null β	f-SAD	0.79	< 0.001
	β-deviations	r-SAD	0.74	< 0.001
	β-deviations	f-SAD	0.80	< 0.001

Regional (γ-), local (α-) and β-diversities were calculated for two spatial scales: small (among 0.01-ha subplots within a 0.1-ha plot) and large (among 0.1-ha plots within an elevational band). Diversity was partitioned following Jost [43] and by weighting each species proportionally by its abundance (i.e. diversity of order 1). Results are also presented for mean null β-diversity and β-deviations (i.e. standardized differences between observed and null β-diversity). Null β-diversity and β-deviations were calculated using two null models, one that randomizes the regional species abundance distribution (r-SAD) and one that fixes it to be identical to the one observed in the empirical data (f-SAD; see Methods). These null models do not maintain the observed number of individuals in each local assemblage (see also Fig. 2). Similar results were obtained using a variety of different β-diversity metrics and null models that constrained the observed number of individuals (S1 and S2 Results).

Elevational gradients in β-diversity persisted after accounting for sampling effects and regional variation in species pools (Table 1; Fig. 2). At both scales and for both null models, the mean null-expected β-diversity decreased monotonically with elevation. Even after accounting for these null-expected gradients, however, β-deviations retained significant relationships with elevation at both scales (Table 1; Fig. 2).

**Fig 3:**
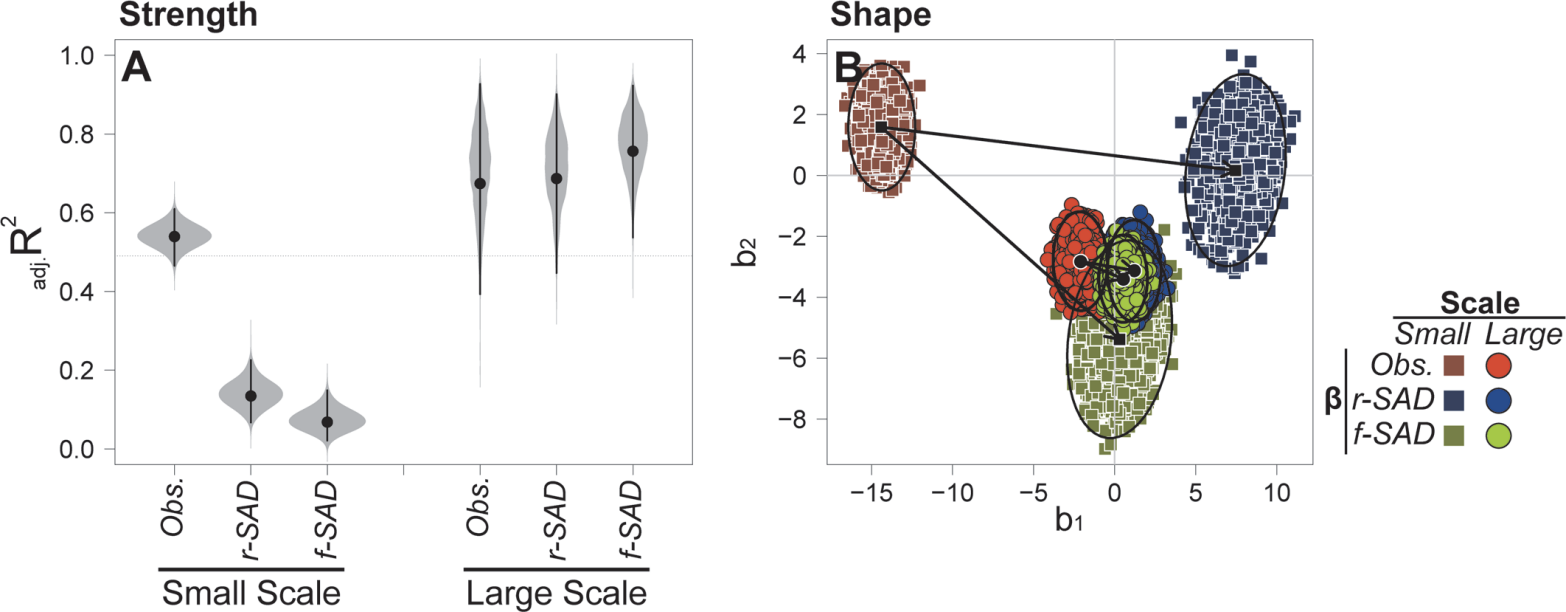
Comparisons of the strength and shape of elevational gradients between scales and between observed β-diversity and β-deviations. β-deviations were calculated using the random SAD (r-SAD) and fixed SAD (f-SAD) null models (see Methods). A) Strength of the gradients measured using adjusted R^2^ values (_adj._R^2^) from cubic regressions between diversity and elevation. Black circles represent original _adj._R^2^ estimates. Grey regions show the distribution of values based on 1,999 bootstrapped regressions. Black lines represent 99% confidence intervals. B) Shape of the gradients measured using standardized regression coefficients. Only the coefficients for elevation (b_1_) and elevation squared (b_2_) are presented. Other coefficients lead to similar conclusions. Black symbols represent original estimates. Black arrows show the change in coefficients between observed β-diversity and β-deviations at a given spatial scale. Black lines represent 99% data ellipses which define confidence regions. Other symbols show the distribution of values based on bootstrapped regressions.

Elevational gradients in β-deviations varied strongly between spatial scales. The strength of the gradient, measured using the proportion of variation in β-deviations explained by elevation (_adj._R^2^), was between 5 and 10 times higher at the large scale relative to the small scale (Table 1; Figs. 2 and 3). At small scales, the variation in β-deviations explained by elevation ranged from 7–14% and was much lower than the explained variation for observed β-diversity (∼54%). At large scales, in contrast, the explained variation for β-deviations ranged from 74–80% and was similar to the explained variation for observed β-diversity (∼73%). In addition, the shape of the gradient was also scale dependent (Table 1; Figs. 2 and 3). At the small scale, β-deviations generally increased with elevation (Fig. 2C), a pattern opposite to the negative relationship for observed x-diversity (Fig. 2B). At the large scale, in contrast, both β-deviations and observed β-diversity showed a mid-elevation peak (Fig. 2E,F), with β-deviations peaking at higher elevations and decaying rapidly above ∼3,700 m.

**Fig 4:**
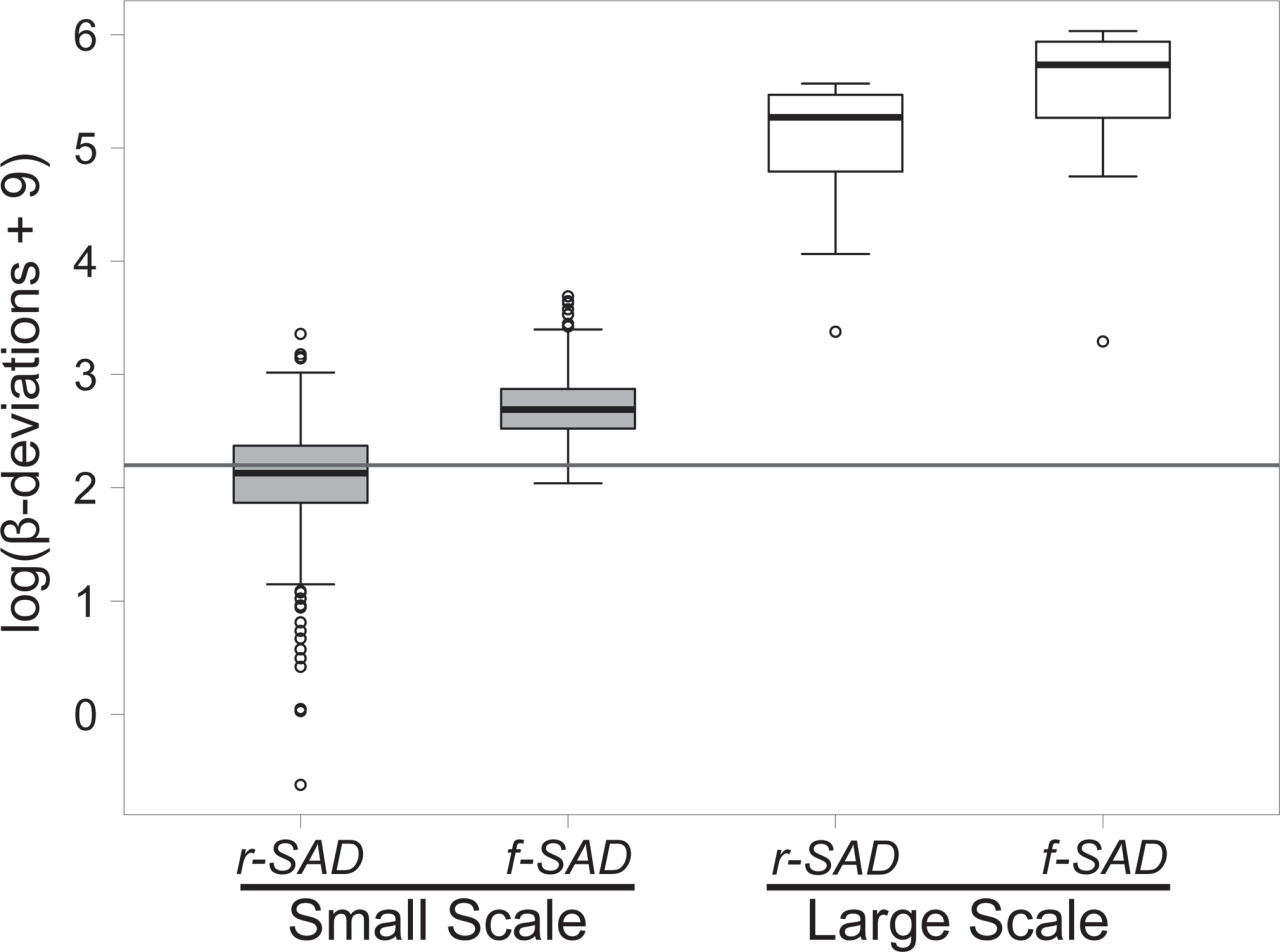
Variation in the overall magnitude of β-deviations between small and large spatial scales. β-deviations were calculated using the random SAD (r-SAD) and fixed SAD (f-SAD) null models (see Methods). The horizontal grey line marks the value of no difference from null model expectations (i.e. β-deviation of zero). β-deviations above the line indicate higher β-diversity than expected by random sampling of individuals from observed species pools. Note that β-deviations are higher at large scales than at small scales (linear mixed-effects model: *t*_276_ < 38.97; *p* < 0.001). In addition, mean β-deviations are statistically different from zero for all combinations of spatial scale and null model (one sample t-tests: |*t*| > 4.77; p < 0.001).

Finally, the magnitude of β-deviations was scale dependent and higher than expected by random sampling. β-deviations were 17 to 19 times higher at the large relative to the small spatial scale (Fig. 4). β-deviations were also typically higher than expected by the null models (Fig. 4). The only exception was at the small scale using the random SAD null model, where β-diversity was slightly lower than null-model expectations.

## Discussion

Our results demonstrate that elevational gradients in β-diversity reflect variation in the strength of local community assembly mechanisms across spatial scales. Specifically, we found that the influence of local assembly mechanisms become stronger and co-vary more tightly with elevation at larger scales. These findings contradict the recent hypothesis that regional variation is species pools alone can account for gradients in β-diversity along broad ecological and biogeographic gradients [11]. Instead, our results suggest that the relative importance of local and regional controls on β-diversity are strongly scale dependent. Together, these results provide some of the strongest insights to date on the relative importance of community assembly mechanisms and regional species pools in shaping species-rich tropical tree communities along elevational gradients.

### Elevational gradients in β-diversity reflect variation in the strength of community assembly mechanisms across spatial scales

We found that the strength of local assembly mechanisms changes systematically along tropical elevational gradients. At small scales, the gradient in observed β-diversity became a weak gradient in β-deviations, suggesting that the gradient in β-diversity at this scale is primarily driven by variation in species pools. Even so, the gradient in β-deviations remained significant, indicating that variation in local assembly mechanisms also contribute to elevational patterns of β-diversity at very small spatial scales. Differences in statistical power can help explain variable results between our analyses and other studies of β-deviations along elevational gradients at small scales. For example, whereas Kraft *et al*. [11] analyzed tropical tree communities using 8 regions along a ∼2,500-m elevational gradient in Costa Rica, our comparable small-scale analyses are based on 440 regions along a ∼4,000-m gradient. Indeed, our chances of finding a significant gradient in β-deviations at small scales using only 8 regions would have been only between 11 and 14% (power analysis results not shown). In addition, Mori *et al*. [14] found a significant elevational gradient in β-deviations at small-scales across low-diversity temperate forests in Japan (∼60 species), a result that parallels our findings in high-diversity tropical forests (∼2,600 species).

At large scales, in contrast, we found a strong gradient in β-deviations similar to the gradient in observed β-diversity. This suggests that the relative contribution of local community assembly processes to elevational gradients in β-diversity is strongly scale dependent. At small scales, variation in local assembly mechanisms might be significant but weak relative to sampling effects owing to variation in species pools. At large scales, on the other hand, local assembly mechanisms vary strongly across elevations, and contribute substantially to elevational patterns of community assembly and β-diversity. Importantly, our results suggest that inferences about assembly mechanisms shaping β-diversity patterns at small scales [11,26] cannot be extrapolated to larger spatial scales. Instead, increases in scale can lead to a reduction in the perceived strength of sampling effects and an increase in the importance of local community assembly processes in shaping elevational gradients in β-diversity.

### Local assembly mechanisms structuring species assemblages are detectable at very small spatial scales, but become stronger at large scales

Our results suggest that the overall strength (magnitude) of local assembly processes varies strongly with spatial scale. After controlling for sampling effects and variation in species pools, we found that β-deviations were 17–19 times larger at large scales compared to small scales. Even so, we found significant deviations from null models even when local assemblages were characterized at very small grain sizes (10×10 m) and separated by at most ∼90 m (i.e. small scale analyses), a pattern also observed in other recent analyses conducted at similarly small spatial scales [11,14,26]. These small-scale deviations could be explained by multiple ecological processes including dispersal limitation [54], small-scale variation in edaphic and topographic characteristics [55,56], and biotic interactions like competition and natural enemy attack at the neighborhood scale [48,57,58]. Many of these processes can also vary with scale, potentially explaining the scale dependency in the magnitude of β-deviations observed in our study. For example, increases in the extent of regions and distances among assemblages can increase environmental heterogeneity and isolation of communities, leading to stronger species sorting or dispersal limitation [59]. Importantly, our results demonstrate that the spatial structure of local assemblages does not result simply from uncorrelated sampling of individuals from species pools [11,23,60], but reflects scale-dependent variation in the strength of community assembly mechanisms.

### Local community assembly mechanisms are weakest in lowland tropical forests and at very high elevations

**Fig 5:**
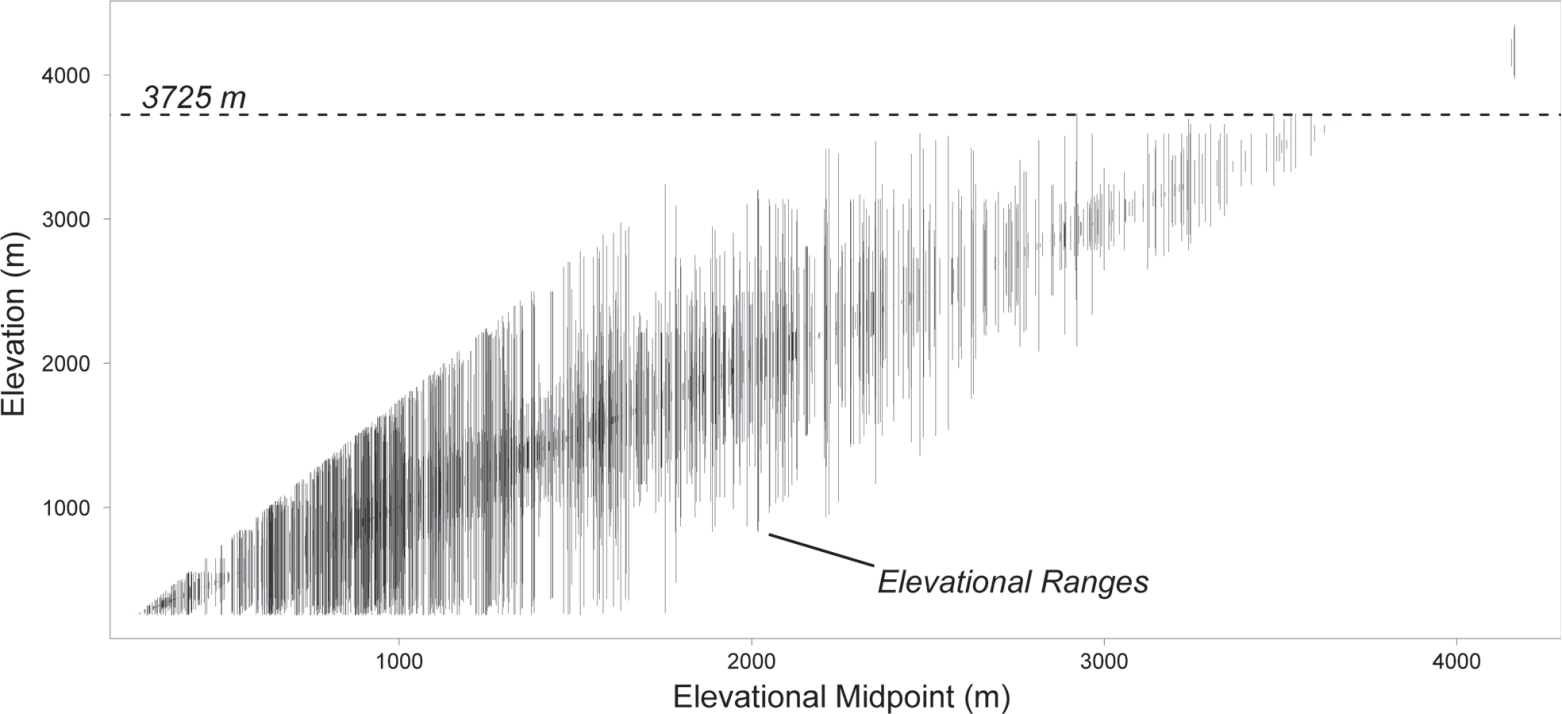
Distributions of 2,668 woody plant species along the elevational gradient. Each vertical line represents the elevational range of a species in the Madidi region. Ranges are defined as the interval between the lowest and highest elevations at which a species was found within the full network of plots. The horizontal dashed line marks the elevation at which there seems to be a break in the continuous turnover in forest composition along the elevational gradient. Above 3,725 m, forests are composed only of 3 woody plant species: *Gynoxys asterotricha*, *G*. *compressissima* and *Polylepis pepei*.

We found that the strength of local community assembly mechanisms generally increased with elevation, but then decreased dramatically for regions above ∼3,700 m. This pattern is very conspicuous at large scales, and subtle at small scales. The observed decrease in the strength of local assembly processes at high elevations coincides with a dramatic shift in the composition of Andean floras. After a gradual replacement of species along the elevational gradient up to approximately 3,700 m, there is a strong shift in species composition such that forests above and below that elevation do not share any species (Fig. 5). This suggests that unique environmental conditions (e.g. temperature) might restrict the membership of species to very high-elevation forests, and potentially also change the dynamics of local community assembly. In contrast, a previous study of lower-diversity temperate forests across a shorter elevational extent (<1,500 m) found a monotonic increase in β-deviations with increasing elevation [14]. A similar pattern was observed across the high-diversity forests in our study, where β-deviations generally increased with elevation below 1,500 m (Fig. 2). Across the entire elevational gradient, however, the signature of local assembly mechanisms structuring forest assemblages appears to be of similar strength in tropical lowlands and at very high elevations.

A variety of local mechanisms could explain the mid- to high-elevation peak in β-deviations [17]. For example, the strength of species-sorting or dispersal limitation may peak at these elevations, creating high dissimilarity among local assemblages. However, we know of no empirical evaluation of changes in environmental heterogeneity or the dispersal ability of species with elevation that could help explain our results. Moreover, mechanisms underlying geographic gradients in β-diversity do not have to vary consistently with the pattern [29], such that similarly low β-deviations at high and low elevations could reflect different mechanisms of community assembly, and these mechanisms can be different from those operating at intermediate elevations where the peak occurs. For example, in a recent comparison of tropical (Bolivia) and temperate (Missouri) regions, Myers *et al*. [29] found similar β-deviations in the two regions. However, β-deviations were more strongly correlated with environmental variables in the temperate region, and more strongly correlated with spatial variables in tropical region. This suggests that the same magnitude of β-deviations may be explained by different mechanisms across biogeographic regions with different species pools. The extent to which elevational gradients in β-deviations reflect shifts in the relative importance of different assembly mechanisms remains an important question for future research in temperate and tropical ecosystems.

## Conclusions

Despite long-standing interest in the ecology, evolution and conservation of elevational-diversity gradients [1–3], surprisingly little is known about elevational patterns and mechanistic drivers of β-diversity, particularly in species-rich tropical regions. Using one of the most well-described elevational gradients of tropical plant diversity, we show that the assembly of communities along broad biogeographic gradients reflects the interplay of local community assembly mechanisms and regional influences owing to variation in species pools. In contrast to the recent hypothesis that variation in species pools alone drives biogeographic gradients in β-diversity [11], we show that variation in local assembly mechanisms contribute strongly to systematic changes in β-diversity across elevations, resulting in a mid-elevational peak in β-diversity. Moreover, we find that the relative importance of community assembly processes is strongly scale dependent. At small scales, local assembly mechanisms are detectable, but random sampling from observed species pools can account for most of the elevational gradient. At large spatial scales, variation in local assembly mechanisms is a dominant force driving changes in β-diversity along elevational gradients. Our study suggests that scale-dependent variation in local community assembly mechanisms, combined with biogeographic variation in species pools, contribute to the origin and maintenance of these iconic and threatened gradients in global biodiversity.

## Supporting Information

S1 Dataset Data for small-scale regions. (XLSX)

S2 Dataset Data for large-scale regions. (XLSX)

S1 Code R functions for null model analyses. (ZIP)

S1 Methods Relative spatial position of plots within large-scale regions and its relationship with elevation. (DOCX)

S2 Methods Patterns of variation in standardized estimates of total species richness for large-scale regions. (DOCX)

S1 Results Analyses using alternative measures of β-diversity. (DOCX)

S2 Results Analyses using null models that maintain the number of individuals per local assemblage. (DOCX)
