# Supplementary material for: Elevational Gradients in β-Diversity Reflect Variation in the Strength of Local Community Assembly Mechanisms across Spatial Scales
Source: PLoS One. 2015 Mar 24;10(3):e0121458. doi: 10.1371/journal.pone.0121458 (PMC4372560; doi:10.1371/journal.pone.0121458)
Supplement: S1 Methods — (DOCX) [file pone.0121458.s004.docx]

# S1 Methods. Relative spatial position of plots within large-scale regions and its relationship with elevation

S1 Text. Creating large-scale regions. Large-scale regions were initially produced by dividing the elevational gradient (254–4,351 m) into 20 equal-range fractions, and grouping together plots falling within each of these fractions. However, these groups differed considerably in the number of plots included and the spatial distribution of plots within a group. Both of these characteristics could influence γ- and β-diversity and needed to be controlled for, so we modified these initial groups of plots to make them comparable across elevations. First, we selected all groups of plots that contained at least 10 plots, reducing the number of groups from 20 to 18. Second, to equalize the number of plots, we selected 10 plots from each of the 18 groups. The selection algorithm searched for combinations of plots that minimized relationships between the spatial configuration of plots within groups and elevation. The spatial configuration of plots within a group was measured using 10 different metrics: the median, standard deviation and sum of all neighbor distances, nearest neighbor distances and minimum spanning tree distances, as well as the range in elevation represented by the plots within a group. After our selection algorithm was applied, none of these metrics had significant linear, quadratic or cubic relationships with elevation (see S1 and S2 Figs.). This guaranteed that relationships between diversity and elevation in large-scale analyses were independent of the spatial configuration of plots within regions. Additionally, ordinary least-squares multiple regressions show these 10 variables cannot explain regional variation in observed β-diversity or β-deviations (_adj._R^2^ ≤ 0.062; *p* ≥ 0.457). These results demonstrate that spatial distribution of plots within regions is not a confounding factor in our analyses. The typical median distance between local assemblages in a large-scale region was 19.3 km (range 1.9 to 41.8 km; S1 Fig.), and the typical range in elevation was 166.2 m (range 117 to 190 m; S2 Fig.).


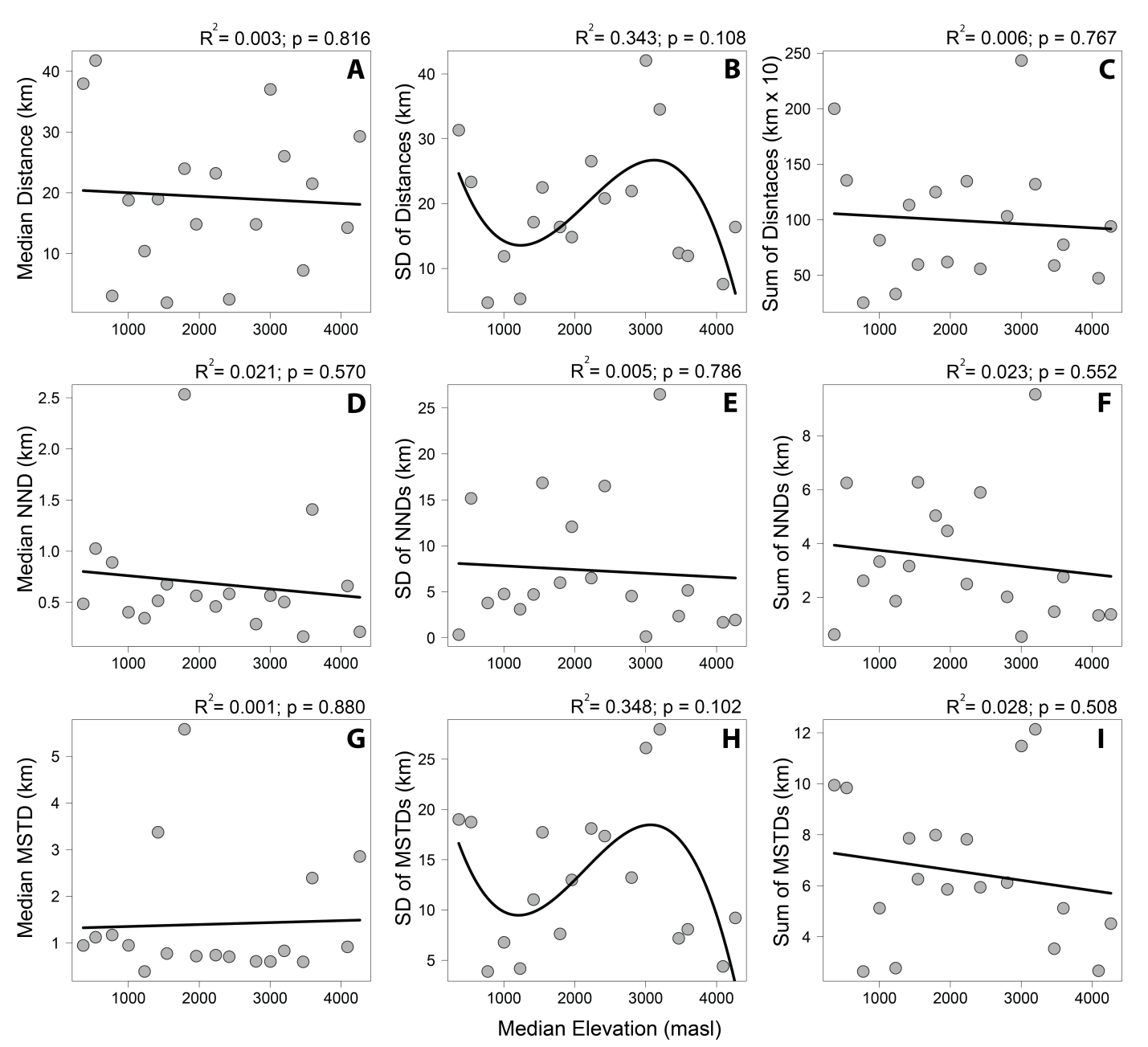


S1 Fig. Relationships between inter-plot distances and elevation for large-scale regions. For each group of plots, we calculated multiple indices of the relative spatial location of plots and then related them against the median elevation of the group. We calculated a total of nine indices reflecting the geographic distances among plots: the median, standard deviation and sum of all neighbor distances (first row), nearest neighbor distances (NND; second row) and minimum spanning tree distances (MSTD; third row). All relationships were non-significant (p > 0.1). See also S2 Fig.

**
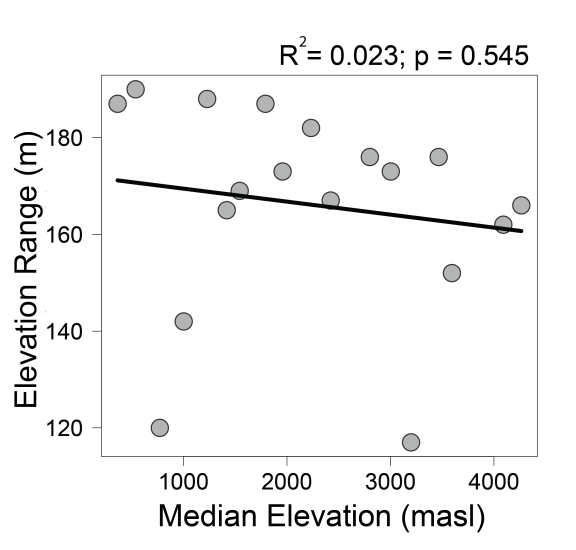
**

S2 Fig. Relationship between within-group range in elevation and elevation for large-scale regions. For each group of plots, we calculated the elevation range of plots in the group and then regressed it to the median elevation of the group. This relationship was non-significant and very weak.
