# Supplementary material for: Elevational Gradients in β-Diversity Reflect Variation in the Strength of Local Community Assembly Mechanisms across Spatial Scales
Source: PLoS One. 2015 Mar 24;10(3):e0121458. doi: 10.1371/journal.pone.0121458 (PMC4372560; doi:10.1371/journal.pone.0121458)
Supplement: S2 Methods — (DOCX) [file pone.0121458.s005.docx]

# S2 Methods. Patterns of variation in standardized estimates of total species richness for large-scale regions

Text S2 – Estimating species richness in large-scale regions by rarefaction and extrapolation. Our set of 10 forest plots in each large-scale region necessarily underestimates the total diversity of species of the elevational bands that each region represents. Also, there are clear differences in number of individuals across regions (S3 Fig.). Because these two potential issues could distort regional patterns of variation in γ-diversity, we investigated elevational gradients in standardized estimates of regional species richness. We used two different approaches for this standardization. First, we used rarefaction, calculating the number of species in each large-scale region expected in a sample of 420 individuals (42 individuals in each of the 10 forest plots in a region). Second, we used extrapolation based on 3 common richness estimates: Chao’s, bootstrapping and Jackknifing method 1(Gotelli and Chao 2013). We found that although the total number of individuals per region varies significantly across the elevational gradient (S3A Fig.), standardized estimates of regional richness have strong positive relationships with the number of species observed in forest plots, and also show the same elevational patterns of variation (S3B-C Fig.). We also found that for most of the elevational gradient, the number of species observed in forest plots represent a constant proportion of the species estimated (S3D Fig.). Only for regions above 4,000 m, the number of species observed in the forest plots is much closer to the estimated number of species in the region. These results suggest that patterns in γ-diversity obtained solely from our 10 forest plots are likely robust, and that additional sampling to improve the coverage of species in large-scale regions would likely not modify our conclusions.

***Additional cited literature***

Gotelli, N.J. and A. Chao. 2013. Measuring and estimating species richness, species diversity, and biotic similarity from sampling data. pp. 195-211 in: S.A. Levin (ed.). Encyclopedia of Biodiversity, 2nd edition. Volume 5. Academic Press, Waltham, MA


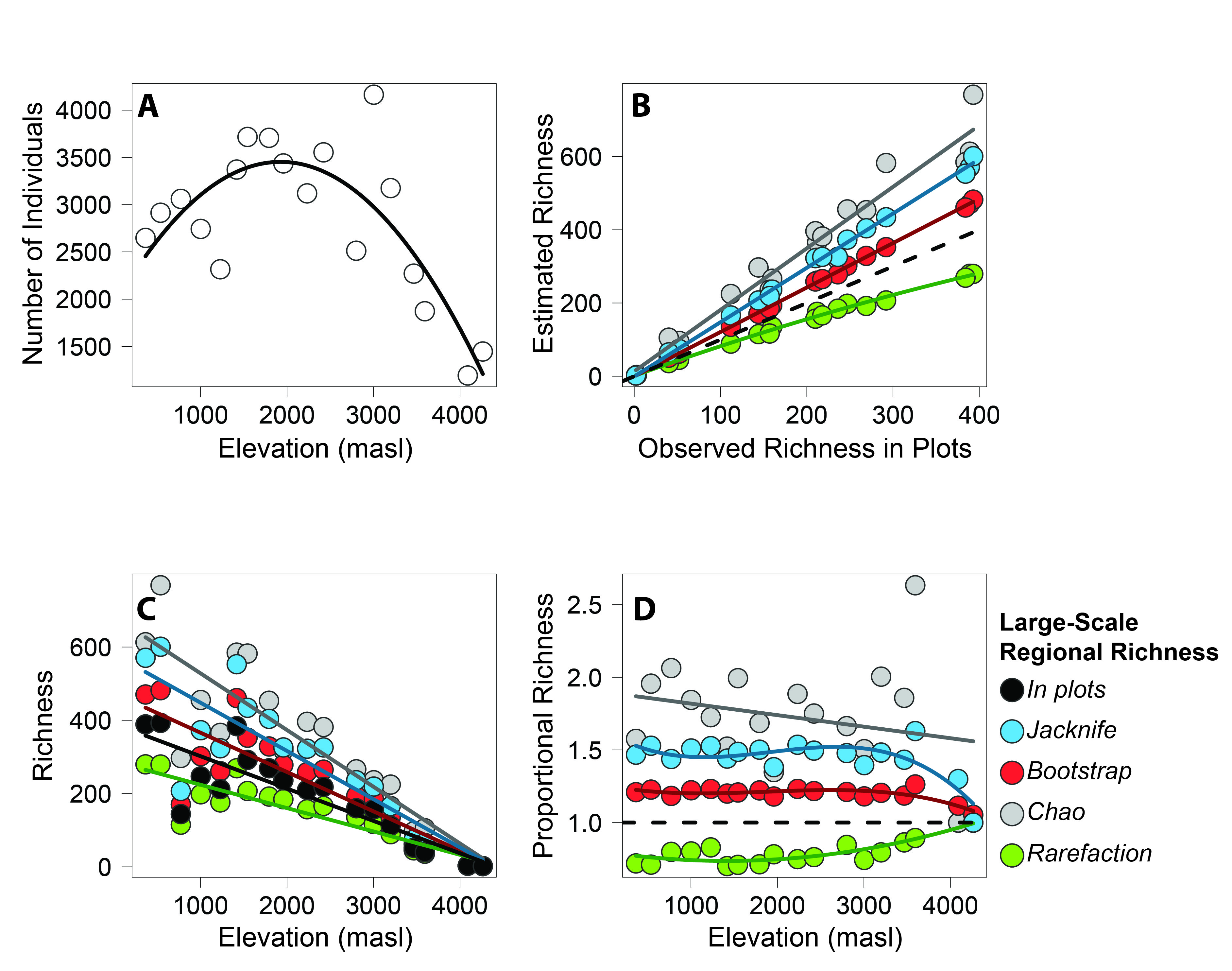


S3 Fig. Variation at large spatial scales of regional abundance and regional species richness estimated by rarefaction or extrapolation. Regional abundance (A); richness (B to D). The dashed line in B represents the 1:1 correspondence line. In D, each estimate of regional species richness was divided by the number of species observed in the ten forest plots. The dashed line here marks the proportion of 1, or of when the observed number of species and the estimated number of species are identical.
