# Supplementary material for: Elevational Gradients in β-Diversity Reflect Variation in the Strength of Local Community Assembly Mechanisms across Spatial Scales
Source: PLoS One. 2015 Mar 24;10(3):e0121458. doi: 10.1371/journal.pone.0121458 (PMC4372560; doi:10.1371/journal.pone.0121458)
Supplement: S1 Results — (DOCX) [file pone.0121458.s006.docx]

# S1 Results. Analyses using alternative measures of β-diversity

S1 Table. Relationships between β-diversity and elevation – results based on alternative measures of β-diversity. Regional β-diversity was calculated for two scales: small (between 0.01ha subplots within a 0.1ha plot) and large (between 0.1ha plots within an elevational band). Results are presented for three alternative measures of β-diversity: (1) mean Bray-Curtis distances among local assemblages, (2) Jost’s (2007) diversity partition by weighting all species equally (i.e., diversity of order 0 or richness), and (3) proportional species turnover (Kraft et al. 2011). Results are also presented for mean null β-diversity and for β-deviations (i.e. standardized differences between observed and null β-diversity). Null β-diversity and β-deviations were calculated based on two null models, one that randomizes the regional species abundance distribution (r-SAD) and one that fixes it to be identical to the one observed in the data (f-SAD). These results are based on null models that do not maintain the empirical number of individuals in each local assemblage (see also S4-S8 Figs.).

| **Spatial Scale** | **Measure** | **Diversity** | **Null Model** | **_adj._R2** | **p-value** |
| --- | --- | --- | --- | --- | --- |
| Small | Mean Bray-Curtis | β |  | 0.552 | < 0.001 |
|  |  | Mean Predicted | r-SAD | 0.723 | < 0.001 |
|  |  |  | f-SAD | 0.586 | < 0.001 |
|  |  | β-deviations | r-SAD | 0.251 | < 0.001 |
|  |  |  | f-SAD | 0.135 | < 0.001 |
|  | ${{}^{q=0}D}_{\beta}$ | β |  | 0.663 | < 0.001 |
|  |  | Mean Predicted | r-SAD | 0.560 | < 0.001 |
|  |  |  | f-SAD | 0.639 | < 0.001 |
|  |  | β-deviations | r-SAD | 0.131 | < 0.001 |
|  |  |  | f-SAD | 0.112 | < 0.001 |
|  | Prop. Species Turnover | β |  | 0.822 | < 0.001 |
|  |  | Mean Predicted | r-SAD | 0.686 | < 0.001 |
|  |  |  | f-SAD | 0.808 | < 0.001 |
|  |  | β-deviations | r-SAD | 0.163 | < 0.001 |
|  |  |  | f-SAD | 0.132 | < 0.001 |
| Large | Mean Bray-Curtis | β |  | 0.883 | < 0.001 |
|  |  | Mean Predicted | r-SAD | 0.911 | < 0.001 |
|  |  |  | f-SAD | 0.879 | < 0.001 |
|  |  | β-deviations | r-SAD | 0.758 | < 0.001 |
|  |  |  | f-SAD | 0.856 | < 0.001 |
|  | ${{}^{q=0}D}_{\beta}$ | β |  | 0.457 | 0.010 |
|  |  | Mean Predicted | r-SAD | 0.693 | < 0.001 |
|  |  |  | f-SAD | 0.849 | < 0.001 |
|  |  | β-deviations | r-SAD | 0.797 | < 0.001 |
|  |  |  | f-SAD | 0.684 | < 0.001 |
|  | Prop. Species Turnover | β |  | 0.826 | < 0.001 |
|  |  | Mean Predicted | r-SAD | 0.769 | < 0.001 |
|  |  |  | f-SAD | 0.928 | < 0.001 |
|  |  | β-deviations | r-SAD | 0.906 | < 0.001 |
|  |  |  | f-SAD | 0.885 | < 0.001 |


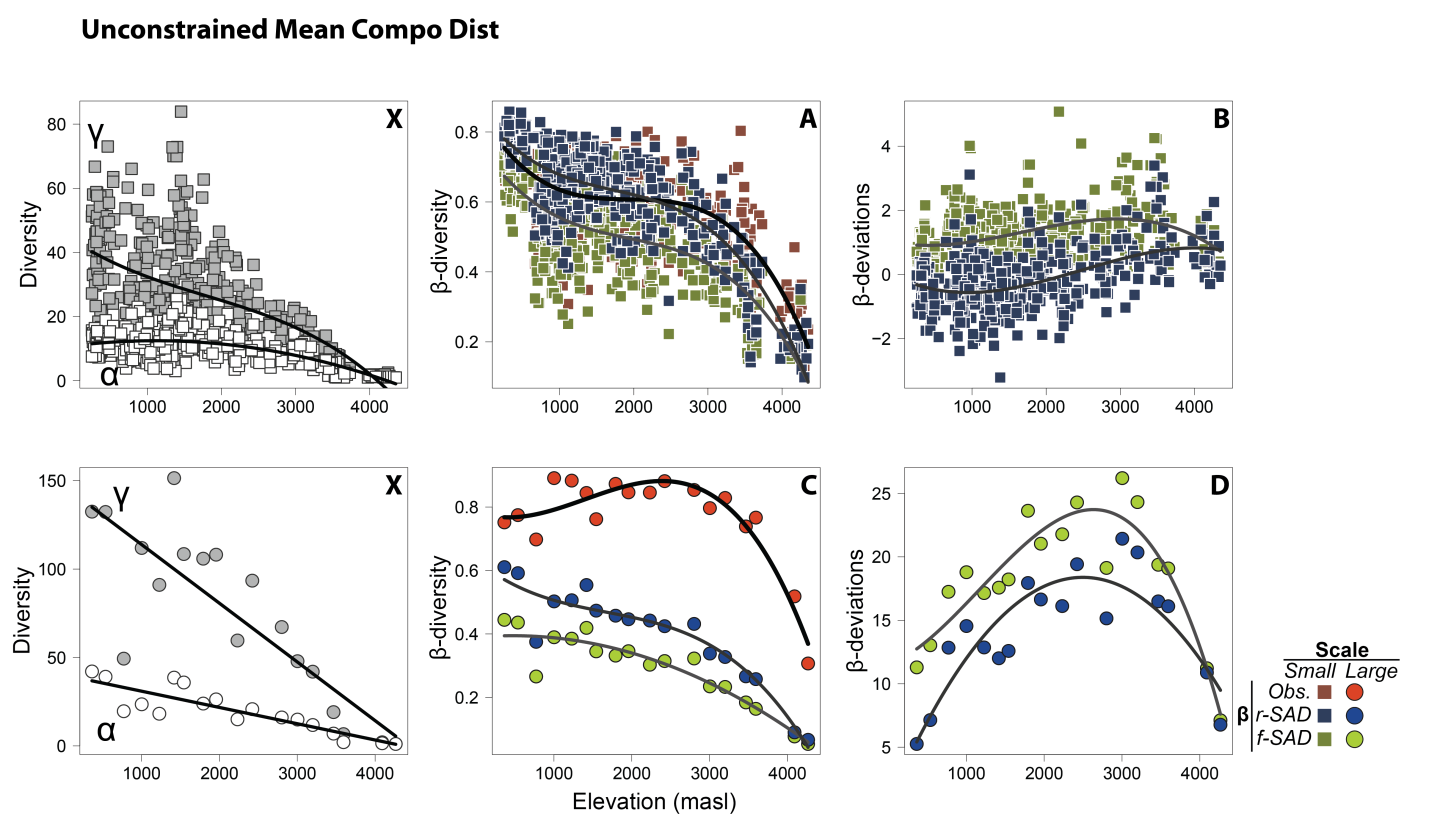


S4 Fig. Elevational gradients in β-diversity at small and large spatial scales based on mean Bray-Curtis distances among all pairs of local assemblages. A) and C) Observed β-diversity and mean null β-diversity. B) and D) β-deviations. Null β-diversity and β-deviations were calculated using the random SAD (r-SAD) and fixed SAD (f-SAD) null models (see Materials and Methods). β-diversity was measured using Jost’s diversity of order one (i.e. exponential Shannon diversity). These results are based on null models that do not maintain the empirical number of individuals in each local assemblage. All relationships were statistically significant (see S1 Table).

**
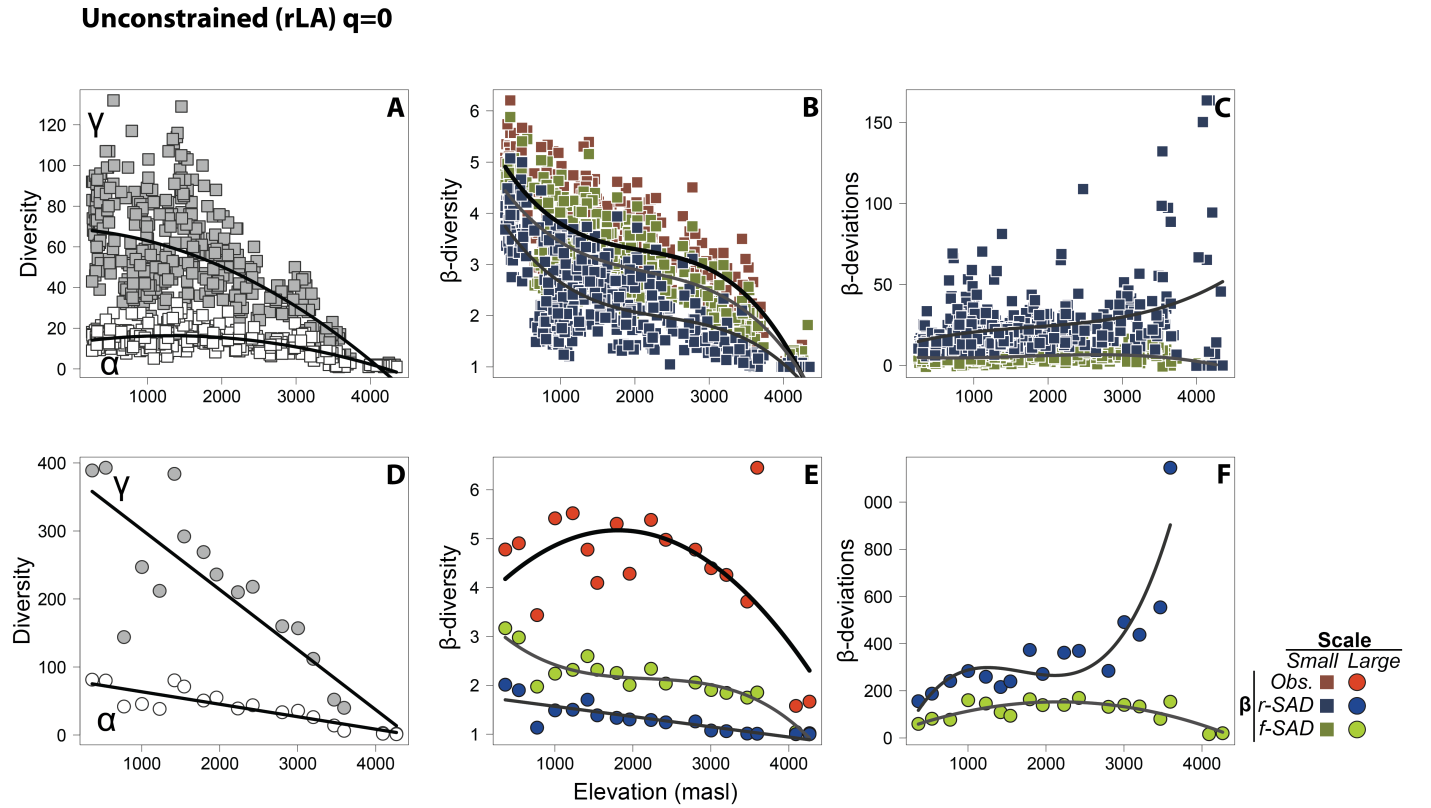
**

S5 Fig. Elevational gradients in diversity at small and large spatial scales based on Jost’s diversity of order zero. A) and D) γ- and α-diversity. B) and E) Observed β-diversity and mean null β-diversity. C) and F) β-deviations. Null β-diversity and β-deviations were calculated using the random SAD (r-SAD) and fixed SAD (f-SAD) null models (see Materials and Methods). β-diversity was measured using Jost’s diversity of order one (i.e. exponential Shannon diversity). These results are based on null models that do not maintain the empirical number of individuals in each local assemblage. All relationships were statistically significant (see S1 Table). The random SAD null model could not produce variation in null β-diversity for sites above 4000 m at large scales; consequently, β-deviations could not be calculated.

**
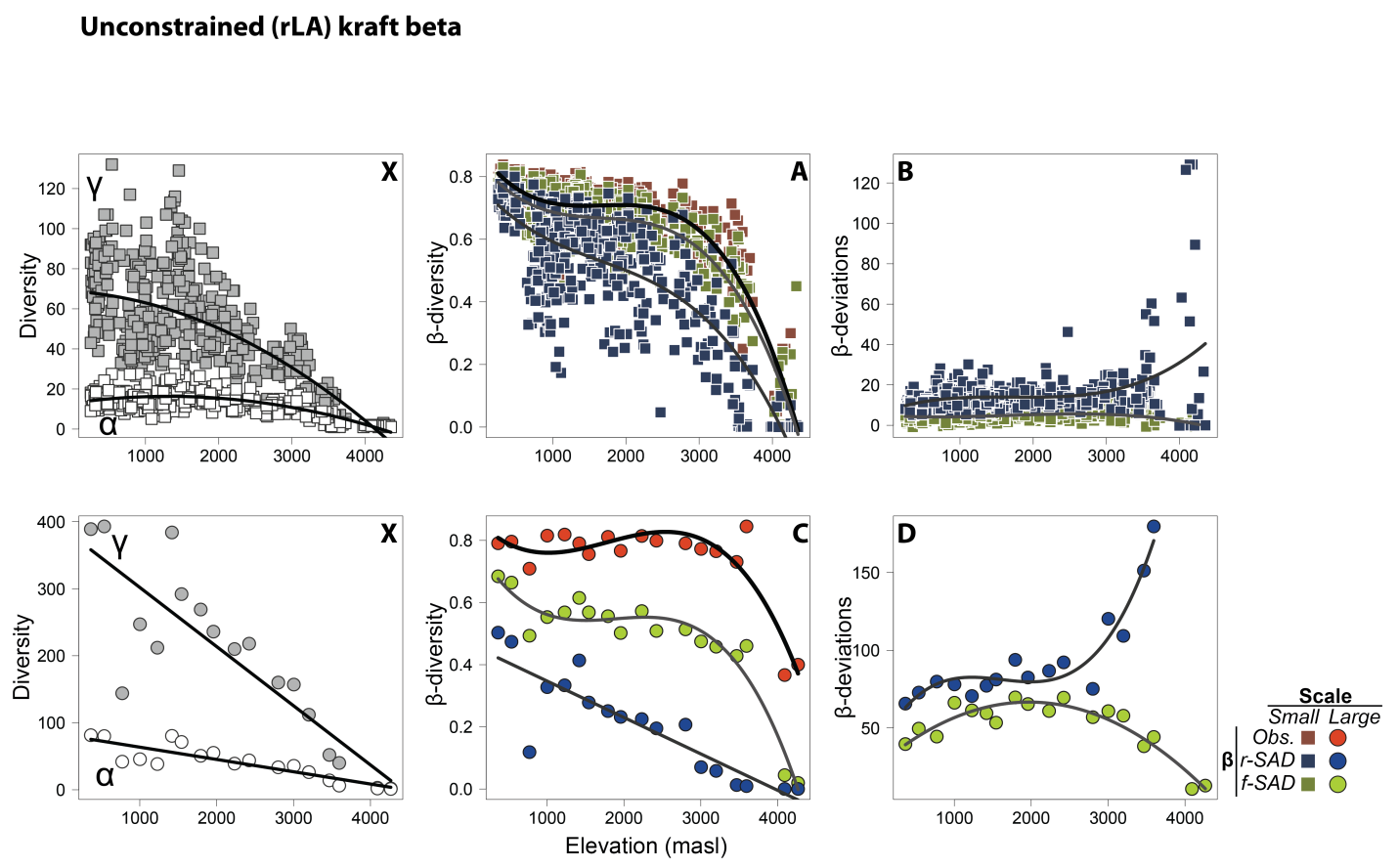
**

S6 Fig. Elevational gradients in β-diversity at small and large spatial scales based on proportional species turnover. Proportional species turnover is calculated as $\beta=1-\bar{\alpha}_{\mathrm{richness}}/\gamma_{\mathrm{richness}}$ (Kraft et al. 2011). A) and C) Observed β-diversity and mean null β-diversity. B) and D) β-deviations. Null β-diversity and β-deviations were calculated using the random SAD (r-SAD) and fixed SAD (f-SAD) null models (see Materials and Methods). β-diversity was measured using Jost’s diversity of order one (i.e. exponential Shannon diversity). These results are based on null models that do not maintain the empirical number of individuals in each local assemblage. All relationships were statistically significant (see S1 Table). The random SAD null model could not produce variation in null β-diversity for sites above 4000 m at large scales; consequently, β-deviations could not be calculated.

**
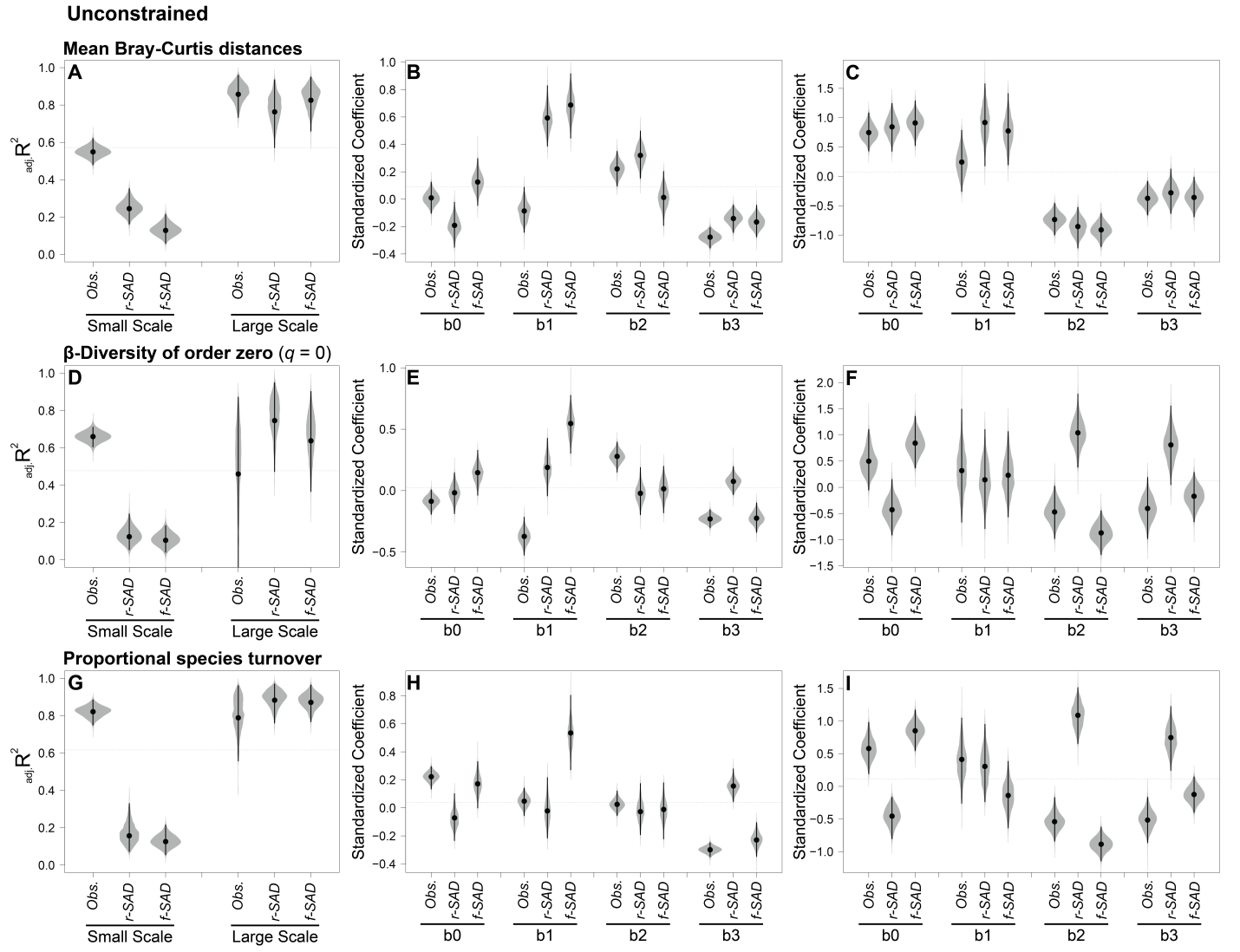
**

S7 Fig. Comparison of strength and shape of the elevational gradients between scales and between observed β-diversity and β-deviations – results based on alternative measures of β-diversity. β-deviations are calculated using the random SAD (r-SAD) and fixed SAD (f-SAD) null models (see Materials and Methods). Results are presented for three alternative measures of β-diversity: (1) mean Bray-Curtis distances among local assemblages (top row), (2) Jost’s (2007) diversity partition weighting all species equally (i.e., diversity of order 0 or richness; middle row), and (3) proportional species turnover (Kraft et al. 2011; bottom row). In panels A, D and G, strength of gradients is quantified using _adj._R2 values of cubic polynomial regressions between diversity and elevation. In panels B, C, E, F, H and I, shape of gradients is quantified using standardized regression coefficients. Coefficients for small scale analyses are presented in the second column, while coefficients for large scale analyses are presented in third column. Black circles represent original _adj._R2 or regression coefficient estimates. Grey regions around circles show variation produced by 1,999 bootstrapped regressions; black lines represent 99% confidence intervals. These results are based on null models that do not maintain the empirical number of individuals in each local assemblage.


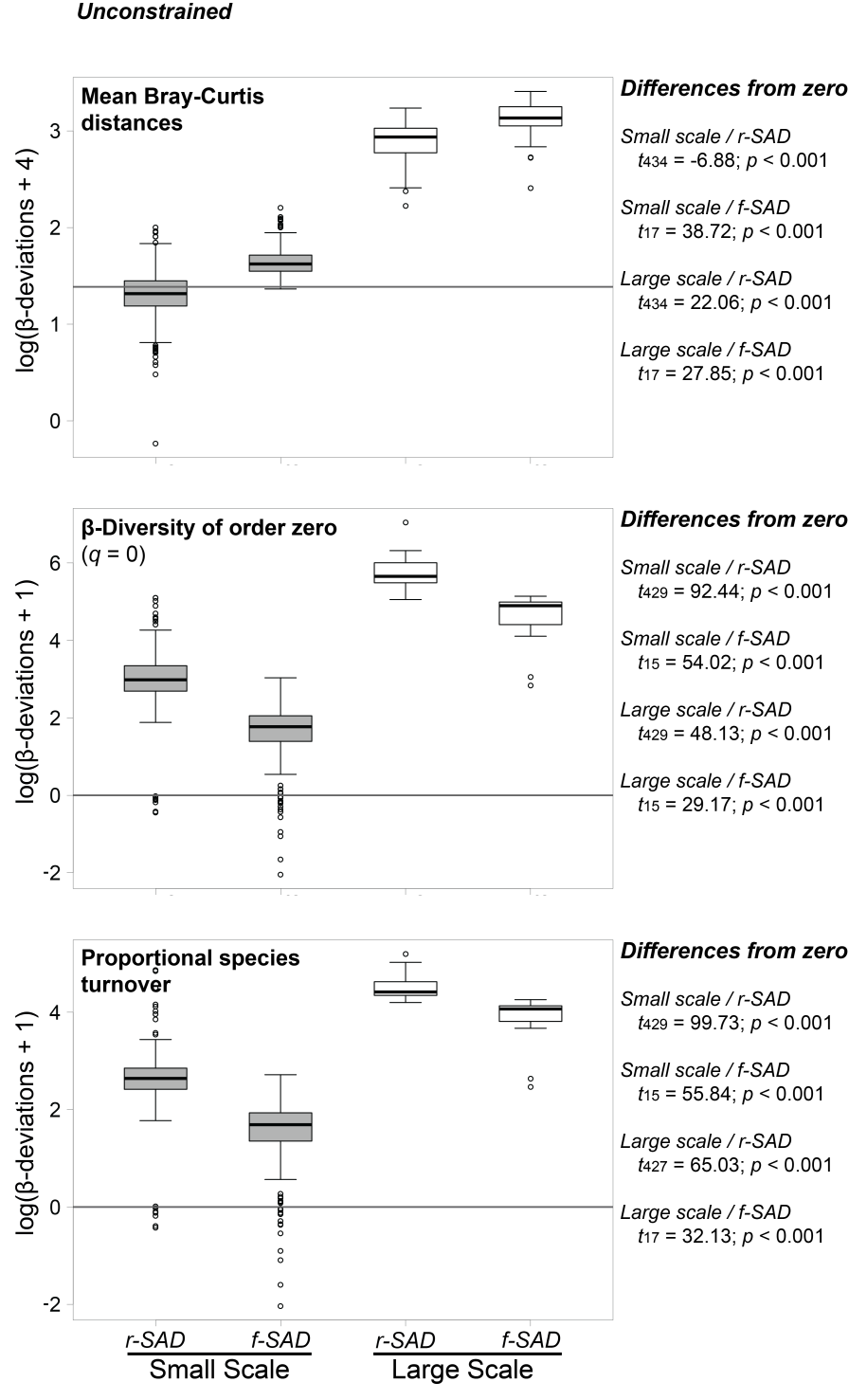
S8 Fig. Differences in magnitude of β-deviations across small and large spatial scales – results based on alternative measures of β-diversity. β-deviations (standardized effect sizes of β-diversity) were calculated based on two null models, one that randomizes the regional species abundance distribution (r-SAD) and one that fixes it to be identical to the one observed in the empirical data (f-SAD; see Materials and Methods). Results are presented for three alternative measures of β-diversity: A) mean Bray-Curtis distances among local assemblages, B) Jost’s (2007) diversity partition weighting all species equally, and C) proportional species turnover (Kraft et al. 2011). Horizontal grey line marks the value of zero, or of no difference from null-model expectations. β-deviations above the line indicate higher β-diversity than expected by random sampling of individuals from the species pool. Linear mixed effects model showed significant differences in β-deviations between spatial scales. Regardless of β-diversity measure, β-deviations are higher at large scales than at small scales (t_277_ ≥ 19.799; p < 0.001). One sample t-test results are also presented in the figure, and demonstrate that mean β-deviations are statistically different from zero for all combinations of spatial scale and null model. These results are based on null models that do not maintain the empirical number of individuals in each local assemblage.
