# Supplementary material for: Elevational Gradients in β-Diversity Reflect Variation in the Strength of Local Community Assembly Mechanisms across Spatial Scales
Source: PLoS One. 2015 Mar 24;10(3):e0121458. doi: 10.1371/journal.pone.0121458 (PMC4372560; doi:10.1371/journal.pone.0121458)
Supplement: S2 Results — (DOCX) [file pone.0121458.s007.docx]

# S2 Results. Analyses using null models that maintain the number of individuals per local assemblage

S2 Table. Relationships between β-diversity and elevation – results based on null models that maintain the empirical number of individuals in each local assemblage. Regional β-diversity was calculated for two scales: small (among 0.01-ha subplots within a 0.1-ha plot) and large (among 0.1-ha plots within an elevational band). Diversity was partitioned following Jost (2007) and by weighting each species proportionally by its abundance (i.e. diversity of order 1). Results are also presented for mean null β-diversity and β-deviations (i.e. standardized differences between observed and null β-diversity). Null β-diversity and β-deviations were calculated using two null models, one that randomizes the regional species abundance distribution (r-SAD) and one that fixes it to be identical to the one observed in the empirical data (f-SAD; see Materials and Methods). These null models are similar to those used by Kraft et al. 2011 and De Cáceres et al. 2012 (see also S9 Fig.).

| **Spatial Scale** | **Diversity** | **Null Model** | **_adj._R^2^** | ***p*-value** |
| --- | --- | --- | --- | --- |
| Small | β |  | 0.543 | < 0.001 |
|  | Mean Predicted β | r-SAD | 0.575 | < 0.001 |
|  |  | f-SAD | 0.543 | < 0.001 |
|  | β-deviations | r-SAD | 0.097 | < 0.001 |
|  |  | f-SAD | 0.071 | < 0.001 |
| Large | β |  | 0.727 | < 0.001 |
|  | Mean Predicted β | r-SAD | 0.745 | < 0.001 |
|  |  | f-SAD | 0.776 | < 0.001 |
|  | β-deviations | r-SAD | 0.739 | < 0.001 |
|  |  | f-SAD | 0.757 | < 0.001 |

***
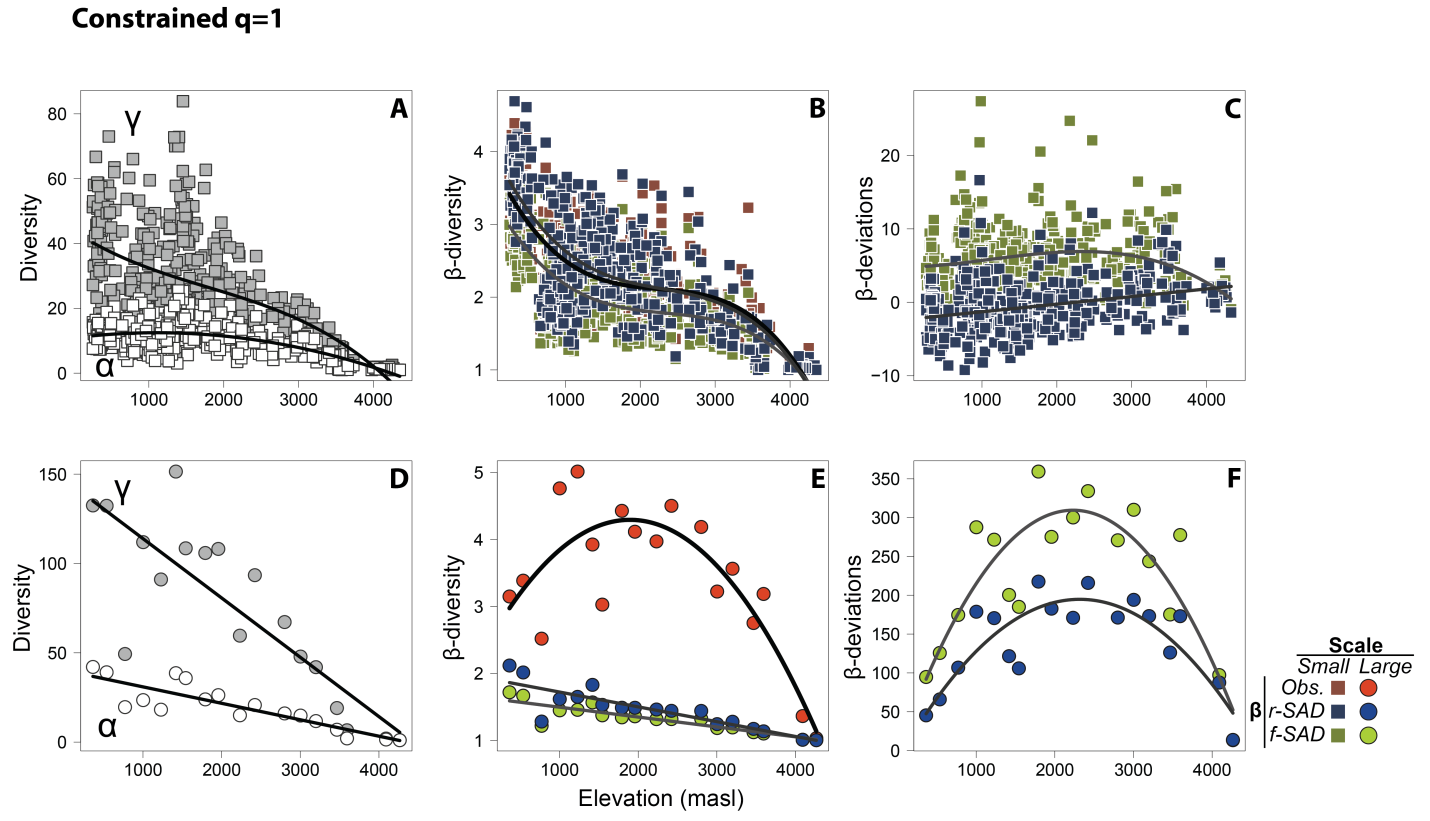
***

S9 Fig. Elevational gradients in diversity at small and large spatial scales – results based on null models that maintain the empirical number of individuals in each local assemblage. A) and D) Regional (γ-) and local (α-) diversity. B) and E) Observed β-diversity and mean null β-diversity. C) and F) β-deviations. Null β-diversity and β-deviations were calculated using the random SAD (r-SAD) and fixed SAD (f-SAD) null models (see Materials and Methods). β-diversity was measured using Jost’s diversity of order one (i.e. exponential Shannon diversity). All relationships were statistically significant (S2 Table). These null models are similar to those used by Kraft et al. 2011 and De Cáceres et al. 2012.

**
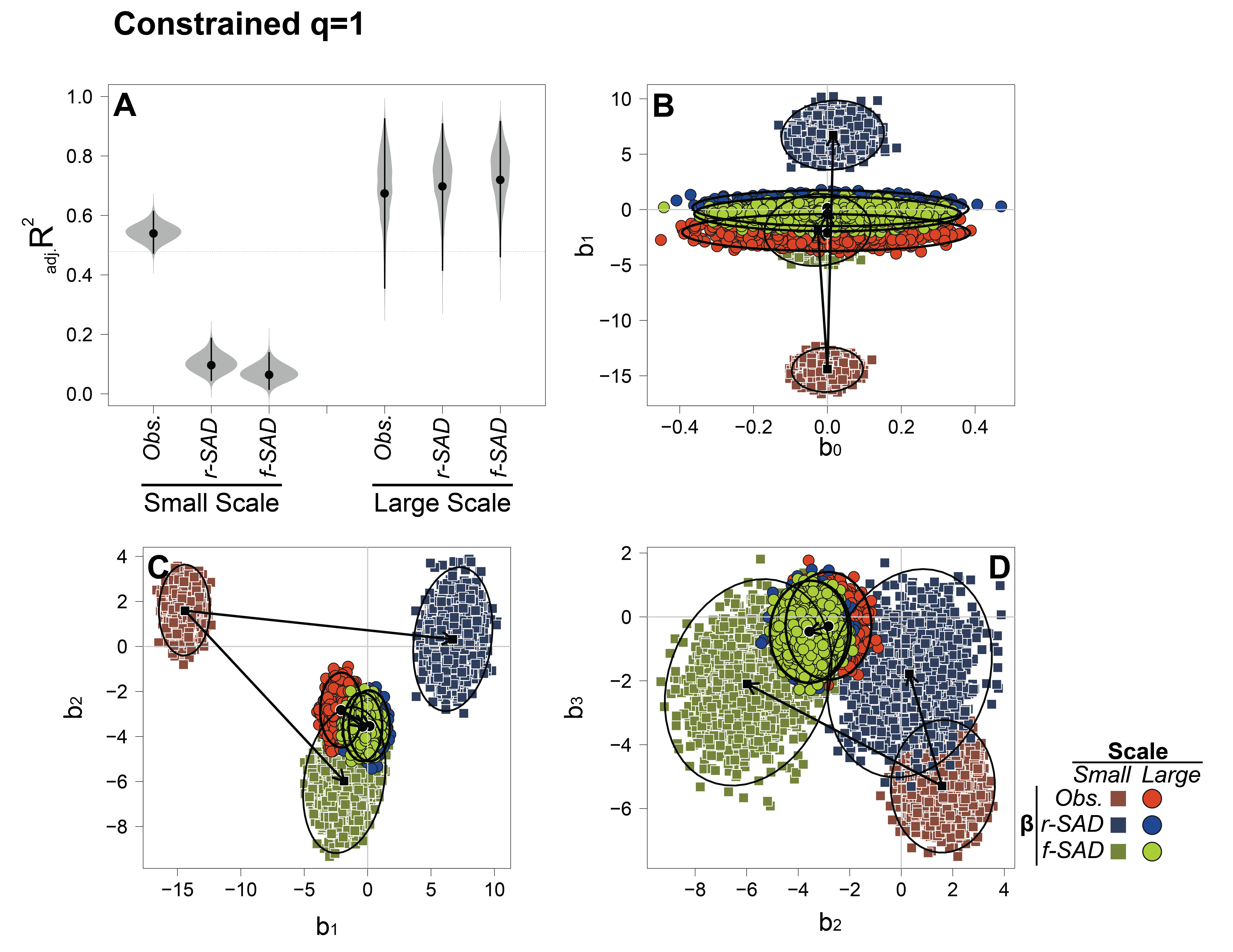
**

S10 Fig. Comparison of strength and shape of the elevational gradients between scales and between observed β-diversity and β-deviations – results based on null models that maintain the empirical number of individuals in each local assemblage. β-deviations are calculated using the random SAD (r-SAD) and fixed SAD (f-SAD) null models (see Materials and Methods). A) Strength of gradients is quantified using _adj._R2 values of cubic polynomial regressions between diversity and elevation. Black circles represent original _adj._R2 estimates. Grey regions around circles show the distribution of values based on 1,999 bootstrapped regressions and black lines represent 99% confidence intervals. B to D) Shape is quantified using standardizes regression coefficients. Black symbols represent original estimates. Black arrows show the change in coefficients between observed β-diversity and β-deviations at a given spatial scale. Colored symbols show the distribution of values based on bootstrapped regressions. Black lines represent 99% data ellipses which define confidence regions. These null models are similar to those used by Kraft et al. 2011 and De Cáceres et al. 2012.

**
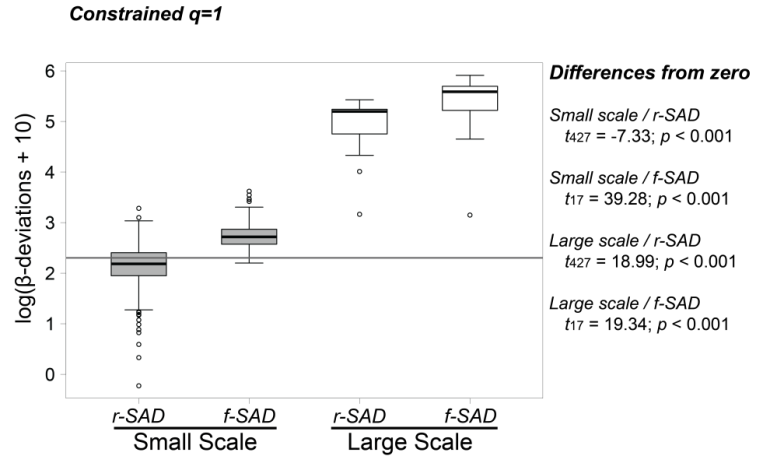
**

S11 Fig. Differences in magnitude of β-deviations across small and large spatial scales – results based on null models that maintain the empirical number of individuals in each local assemblage. β-deviations (standardized effect sizes of β-diversity) were calculated based on two null models, one that randomizes the regional species abundance distribution (r-SAD) and one that fixes it to be identical to the one observed in the empirical data (f-SAD; see Materials and Methods). Horizontal grey line marks the value of no difference from null model expectations. β-deviations above the line indicate higher β-diversity than expected by random sampling of individuals from species pools. A linear mixed effects model showed that β-deviations are higher at large scales than at small scales (t_276_= 39.400; p < 0.001). Also, one sample t-test results demonstrate that mean β-deviations are statistically different from zero for all combinations of spatial scale and null model. These null models are similar to those used by Kraft et al. 2011 and De Cáceres et al. 2012.
